# Annotations

**Published:** 1902-02-01

**Authors:** 


### ANNOTATIONS.

### Theories about Cancer.

Pending the auspicious moment when some millionaire will found an endowment for the investigation of cancer we devote ourselves to the interesting game of guessing, and the latest guess is that cancer has something to do with drains. It is found by Dr. Harold Mason, of Leamington, after investigating a considerable number of cases of cancer, that in regard to upwards of one fourth of all the houses in which cancer had occurred, complaints of the drainage had been made at the offices of the sanitary authority. Moreover, cancer is by no means evenly distributed over the whole area; its distribution is "patchy." It was, perhaps, not very common to find more than one case of cancer in any one house, but it was very common to find the disease occurring in several houses in the same row or on the same side of a street, and especially in contiguous houses, while there were many whole streets in which no deaths from cancer had taken place. Again, a large proportion — 17·5 per cent. — of the houses in which cancer had occurred were the end houses of rows or corner houses of streets, or the houses on either side of court entrances, the places where faults in drains are most likely to arise, and especially was it found to be common for two or more houses adjacent to each other to be "cancer houses," suggesting some common cause presumably in the soil on which they stood. These facts, we are assured, point to the conclusion that whatever the ultimate cause may be, cancer will be found to be due to some germ whose habitat is a sewage-contaminated subsoil, and we are told that this will explain why cancer falls so predominantly on the alimentary system since the food is kept in the cellars; why, as people grow older and are more confined to the house, they become more subject to cancer; why women whose domestic duties keep them longer exposed to any poisonous influences which may arise from the soil on which the house is built, suffer more than men; and lastly, it is even suggested that the very shape of female garments would tend to concentrate these morbific influences upon those female organs which, as we know, are so sadly apt to suffer from the disease. All this is very ingenious, and so far as it is suggestive it is useful, but, much as we should have liked to throw another stone at our old enemy the drains, we are afraid it is but guesswork.

### "A Fee will be Paid by the Company."

This is one of the little inducements offered to medical practitioners when they are asked by assurance companies to spend their time and rack their brains, and also, be it said, to incur the risk of offending a well-paying patient, by giving what the said companies are pleased to call a "confidential report" upon the health prospects of some one whom they have attended. The doctor spends his energies in full confidence of receiving his due reward. He deals honestly with the company believing that so wealthy a company will deal honestly with him; and what does he get in return? Well, that depends upon the company. Some honourably pay their guinea or even more for services rendered, others accept the services but send back only half the fee! Now in regard to this contemptible trick we will not say that the company diddles the doctor, but rather that the doctor shows his folly in trusting to such vague promises. We do say, however, that when people go to insure their lives, if they wish to make a safe investment, they will do well to make certain that they are not dealing with a company which scamps its medical examinations. Doubtless many people regard life assurance merely as a provision against early and unexpected death. But the great majority of those who insure their lives live on; their premiums are an investment bottled up for thirty or forty years; they stake their all on the stability of the company, and this stability (and in "mutual" offices the bonuses to be received) depends absolutely upon the amount of care the company takes during a long series of years in rejecting bad lives. No office which persistently discourages proper medical reports for the sake of a trumpery saving in fees can be successful in the long run, and it is what will happen "in the long run" that is chiefly of interest to the insurer. Any company can receive dividends; the question is which company will be able, thirty or forty years hence, to pay claims? And certainly that will not be the one that, by cutting down medical fees, fills up its books with "bad lives."

### An Operation's Good Repute.

A question has been raised whether in deciding upon a line of treatment one ought to pay any regard to the reputation of the operation which is proposed. In discussing the propriety of performing what he called "tremendous operations upon practically hopeless cases" of cancer, Sir William Banks lately said that he had heard it argued that they should be done even if there was but a hundred-to-one chance for the patient. But he entirely refused to subscribe to such a doctrine. For one who recovered ninety-nine would perish from the operation or from speedy return of the disease, and the disasters to these ninety-nine would probably affect the minds of several hundreds of friends and relations who would hear about their unhappy fate. Thus the operation would be prejudiced in the eyes of the public, and many would be terrified out of submitting to an early operation by which they might be saved. Referring more particularly to cases of cancer of the breast, Sir William Banks said that when a woman discovers a lump in her breast the unfortunate ends of persons whom she has known or heard about occur to her, and so frighten her, that she conceals her complaint until it is too late for any good to be done. In such cases as those suggested, cases in which the chances are really a hundred to one against success, although a patient may be justified in urging that such chance as there is should be given her, there is no doubt much to make the surgeon draw back from participating in such a venture. But in the ordinary run of cases, where the chances are more evenly balanced and where the operation in question, although severe and even dangerous, does afford such a prospect as to make a surgeon feel that, so far as his patient is concerned he is justified in undertaking it, we fancy most surgeons would take but little thought for the credit of the operation, and would consider the whole problem as a question of what is best for the individual before them; and this we think is the only proper course.

